# A Prospective Study on the Effectiveness of Sensory and Sub-sensory Stimulation Amplitudes Using eCoin® Implantable Tibial Nerve Stimulation in Reducing Urgency Urinary Incontinence Episodes and Enhancing Quality of Life

**DOI:** 10.7759/cureus.81121

**Published:** 2025-03-24

**Authors:** Vincent Lucente, Shelby Morrisroe, William Schiff, Nicole Barber

**Affiliations:** 1 Obstetrics and Gynecology, St. Luke's University Health Network, Allentown, USA; 2 Urology, Genesis HealthCare Partners, Torrance, USA; 3 Urology, Urology Associates of Central California Medical Group, Inc., Fresno, USA; 4 Regulatory and Clinical Affairs, Valencia Technologies, Valencia, USA

**Keywords:** ecoin, implantable tibial nerve stimulation devices, neurostimulation, overactive bladder, sensory stimulation amplitudes, urgency urinary incontinence

## Abstract

Introduction

Urgency urinary incontinence (UUI), an important subset of overactive bladder (OAB), manifests with symptoms such as urgency, frequency, and incontinence, severely impacting quality of life. Neuromodulation therapies, including sacral nerve stimulation (SNM), percutaneous tibial nerve stimulation (PTNS), and implanted tibial nerve stimulation (ITNS), are FDA-approved for treating UUI. Traditional neuromodulation involves sensory and motor response evoking amplitudes, but emerging evidence suggests that sensory and sub-sensory settings might enhance treatment outcomes by influencing brain activation.

Aim

This study investigates the efficacy of sensory and sub-sensory programming of the eCoin® ITNS (Valencia Technologies Corporation, Valencia, California, USA) in reducing UUI episodes. The eCoin ITNS is a fully implantable device providing low-duty cycle tibial nerve stimulation.

Methods

The ESSENCE (Effectiveness of Sensory and Sub-sensory Stimulation Amplitudes Using eCoin Implantable Tibial Nerve Stimulation in Reducing Urgency Urinary Incontinence Episodes) study was conducted as a double-blind, randomized, controlled trial, and 36 subjects with UUI across five U.S. centers were enrolled, aiming to evaluate changes in UUI episodes and quality of life over three months. Participants were randomized to sensory or sub-sensory stimulation groups, with the sensory group activated to the amplitude at which stimulation was first felt and the sub-sensory group set 25% below this threshold. UUI episodes were recorded using three-day voiding diaries, and quality of life was assessed via the Overactive Bladder Symptom Quality of Life Questionnaire (OABq) to assess the primary endpoint of reduction from baseline in the number of UUI episodes per day on the three-day voiding diary.

Results

Results demonstrated a mean reduction in UUI episodes of 2.1 per day for the sensory group and 2.73 per day for the sub-sensory group from a pooled baseline of 5.53. Both groups reported improvements in health-related quality of life (HRQL) and patient satisfaction. These findings align with previous studies on SNM, demonstrating both sub-sensory and sensory settings are effective, potentially enhancing patient comfort and device longevity.

Conclusion

The ESSENCE study indicates that both sensory and sub-sensory amplitude settings of eCoin ITNS show a reduction in UUI episodes, improve quality of life, and increase patient satisfaction, offering the potential for optimizing neuromodulation therapies.

## Introduction

Overactive bladder (OAB) presents bothersome urinary symptoms like urgency, frequency, and incontinence, affecting 7-27% of men and 9-43% of women [1]. Urgency urinary incontinence (UUI), which affects approximately one-third of patients with OAB, adversely impacts the quality of life [2,3]. FDA-approved neuromodulation treatments that provide relief for UUI include sacral nerve stimulation (SNM), percutaneous tibial nerve stimulation (PTNS), and implanted tibial nerve stimulation (ITNS). Traditionally, neuromodulation treatments have been programmed with amplitude settings that evoke a sensory and motor response [4]. Prior studies suggest that adjusting stimulation settings, particularly at sensory and sub-sensory levels, influences brain activation and potentially enhances treatment outcomes [5,6]. This research focuses on UUI, exploring the impact of sensory and sub-sensory programming using the FDA-approved eCoin® ITNS (Valencia Technologies Corporation, Valencia, California, USA) to reduce UUI episodes, aiming to optimize therapy effectiveness and extend the longevity of the device.

eCoin ITNS, the FDA-approved fully implantable tibial neurostimulator, is a coin-sized device emitting a dome-shaped electrical field for low-duty cycle tibial nerve stimulation, effectively treating UUI. The device implantation is conducted under local anesthesia in the lower leg. Once activated, it delivers automatic 30-minute treatment sessions requiring no patient intervention. In this randomized controlled trial (RCT), two programming methodologies (sensory and sub-sensory) were explored to assess their impact on reducing UUI episodes. This has been previously studied by Elterman et al. for SNM, which concluded that sub-sensory and sensory amplitude settings demonstrated a similar reduction in UUI episodes; however, there is little evidence on the effect of sub-sensory amplitude settings on UUI symptoms, particularly regarding tibial nerve stimulation [6]. The study aims to explore the effect of sensory and sub-sensory amplitude settings on UUI episodes and quality of life as measured by voiding diaries and surveys at eight and 12 weeks.

## Materials and methods

Study overview

ESSENCE (Effectiveness of Sensory and Sub-sensory Stimulation Amplitudes Using eCoin Implantable Tibial Nerve Stimulation in Reducing Urgency Urinary Incontinence Episodes) is a double-blind, randomized, controlled, prospective, multicenter study (ClinicalTrials.gov: NCT05882318) to evaluate the effectiveness of eCoin tibial nerve stimulation programmed to sensory and sub-sensory levels in individuals suffering from UUI. The primary endpoint of the trial evaluated changes in UUI episodes from baseline through three months of ITNS therapy, measured by voiding diaries and secondary endpoint analysis of patient-reported quality of life outcomes. The study protocol and informed consent documentation were reviewed and approved for all five sites by the central Institutional Review Board (IRB), Western Copernicus Group (WCG) IRB, with the tracking number 20231911. The study was conducted across five centers in the United States. The first subject was enrolled in the study on 16 June 2023. The last patient follow-up visit occurred on 14 May 2024. Written informed consent was obtained from all study participants included in the study. Enrolled subjects were required to meet all inclusion criteria and no exclusion criteria. Key inclusion criteria included adults above 18 years old with daily UUI with a predominantly urgency component. All subjects must be intolerant of, or show an inadequate response to, at least one second or third-line therapy, i.e., drug or peripheral nerve percutaneous neurostimulation, prior to enrollment. Subjects should be appropriate for eCoin therapy based on the US FDA-approved instructions for use (IFU) requirements. Key exclusion criteria included subjects with predominantly stress incontinence, clinically significant bladder outlet obstruction (evaluated by post-void residual (PVR) results and subject’s medical history), neurogenic bladder dysfunction, abnormal PVR, recent anti-stress incontinence operation, bladder pain syndrome, peripheral neuropathy, lower leg varicosities, venous insufficiency with skin changes or pitting edema near the ankle, or peripheral arterial disease. OAB medications should be washed out at least two weeks prior to baseline while prior PTNS and onabotulinumtoxinA patients should have washout periods of one and nine months, respectively. The list of inclusion and exclusion criteria is available on the ClinicalTrials.gov page for the NCT05882318 study. Subjects were expected to avoid OAB therapies until the primary endpoint.

**Figure 1:**
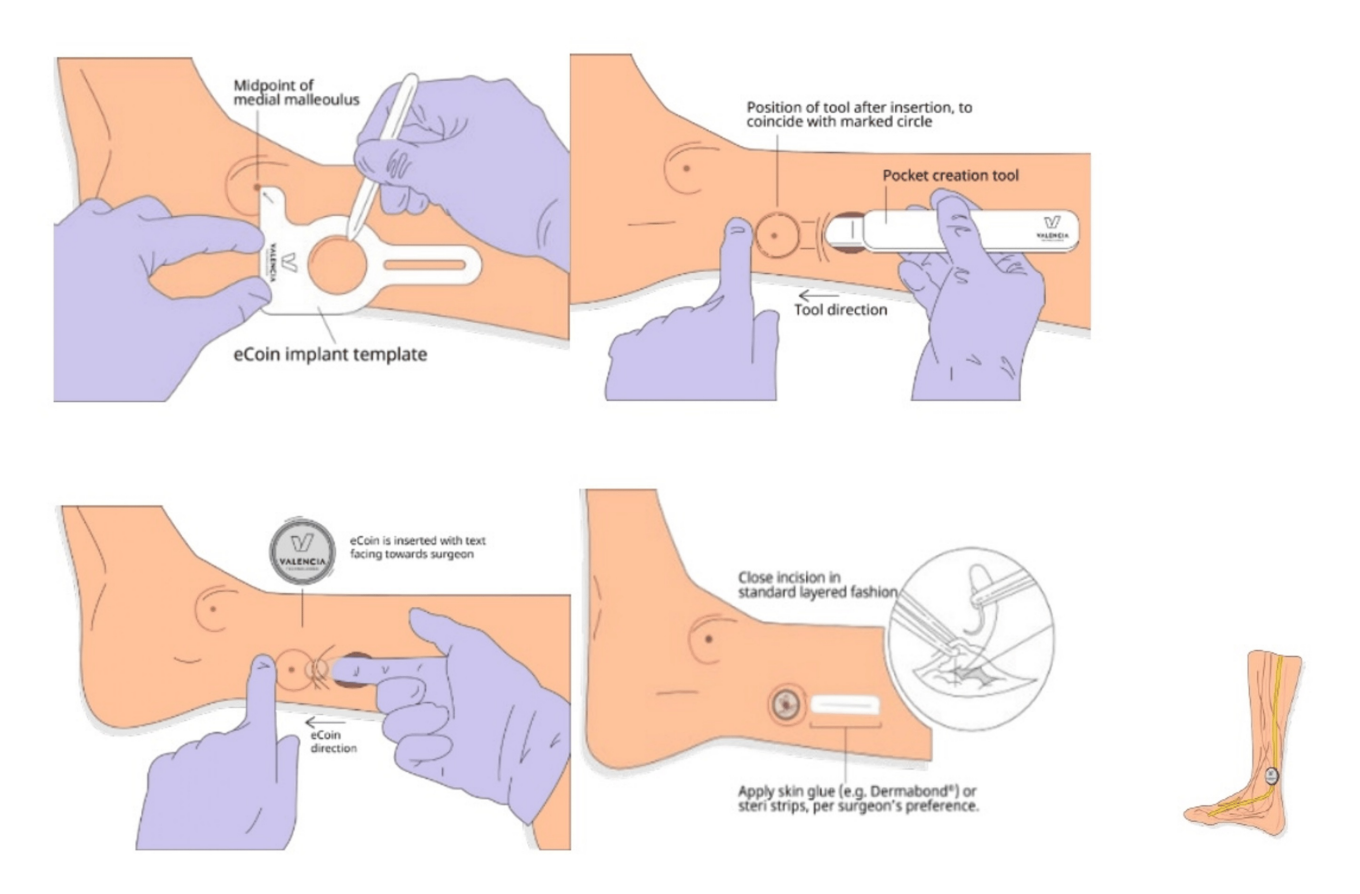
The Implantation Process of eCoin Credit: The images are the property of Valencia Technologies, and permission was obtained for their use.

After verification of eligibility, ITNS was implanted subcutaneously using only local anesthetic in the medial lower leg above the fascia, and an incision site healing check was performed approximately two weeks later. The illustrations of the device implantation process can be seen in Figure 1. After approximately four weeks, subjects had an activation visit where the device was activated (turned on) by a Field Clinical Engineer. During the activation visit, subjects were randomized to a sensory or sub-sensory group. Randomization assignments were in a 1:1 parallel assignment model according to a Randomization Allocation Spreadsheet in order of activation.

The ITNS amplitude level ranges from 0.5 to 15 mA. The sensory group was activated to the amplitude where the subject first felt stimulation. The subject's sensory threshold was determined by turning the device on to 0.5 mA and increasing the amplitude by one level until the subject reported sensation. If the subject did not feel any sensation, they were set to 8 mA and considered a protocol deviation. The sub-sensory group was activated to an amplitude approximately 25% below their first sensory level, according to Table 1. Subjects who did not have sensory at the highest amplitude were set at 11 mA. Any subjects experiencing sensory with amplitudes set to the lowest setting 0.5 mA were considered a protocol deviation. Subjects were required to remain in their randomized treatment arm and not undergo reprogramming until the three-month primary endpoint was reached. After reaching the three-month primary endpoint, subjects were unblinded and reprogramming of amplitude was performed at the request of the investigator or patient, and subjects were followed for an additional month to a total of four months post activation.

**Table 1:** Amplitude Settings for the Sub-sensory Group

First Sensory Threshold	Amplitude Level Set for Sub-sensory Group
0.5 mA	Protocol Deviation, set 0.5 mA
1 mA	0.5 mA
2 mA	1 mA
3 mA	2 mA
4 mA	3 mA
5 mA	4 mA
6 mA	5 mA
7 mA	5 mA
8 mA	6 mA
9 mA	7 mA
10 mA	8 mA
11 mA	8 mA
12 mA	9 mA
13 mA	10 mA
14 mA	11 mA
15 mA	11 mA
No sensation	11 mA

 Changes in UUI episodes were measured using a paper three-day voiding diary to complete within the seven days prior to the baseline, two-, three-, and four-month post-activation visits. Quality of life outcomes were measured using the health-related quality of life (HRQL) section of the Overactive Bladder Symptom Quality of Life Questionnaire (OABq) [7]. Subjects also completed a patient satisfaction survey [8].

The ESSENCE RCT is a double-blinded study. The principal investigator, all clinical staff, and the subject were blinded to the subject's treatment assignment until the primary endpoint visit was completed three months post-activation.

Statistical considerations

The minimum sample size required for this study is 26 subjects (13 per arm) to assess the primary objective of reducing UUI episodes per day on a three-day voiding diary after three months of ITNS in each randomization group. This sample size assumes a 10% attrition rate and a 5% rate of programming-related protocol deviations. A minimum of 32 subjects (16 per arm) were enrolled to ensure that 26 subjects (13 per arm) completed the study in the per-protocol analysis. The actual number enrolled exceeds the minimum of 32 subjects. Based on a confidence interval (CI) using a t distribution, a two-sided type I error rate of 0.05, and a standard deviation of 2, precision was calculated for a range of sample sizes using the PASS (Power Analysis and Sample Size Software) One Mean Module with Tolerance Probability (NCSS, LLC, Kaysville, Utah, USA). A sample size of n=16 per arm will have a precision of 1.07. Accounting for an assumed 15% attrition due to withdrawal or deviations from the assigned therapy group, a sample size of 13 per arm has a precision of 1.21. This precision is less than the presumed treatment effect of 1.3 UUI episodes per day.

The sample size was calculated based on the primary outcome measure of UUI reduction at three months. It was not determined with assumptions regarding attrition beyond the three-month endpoint. Following the three-month visit, subjects had the option to undergo reprogramming, therefore the four-month data is presented as a pooled measurement, as the treatment arms were no longer valid, while still providing insights into the efficacy and safety of the subjects.

## Results

Subject demographics and disposition

**Figure 2:**
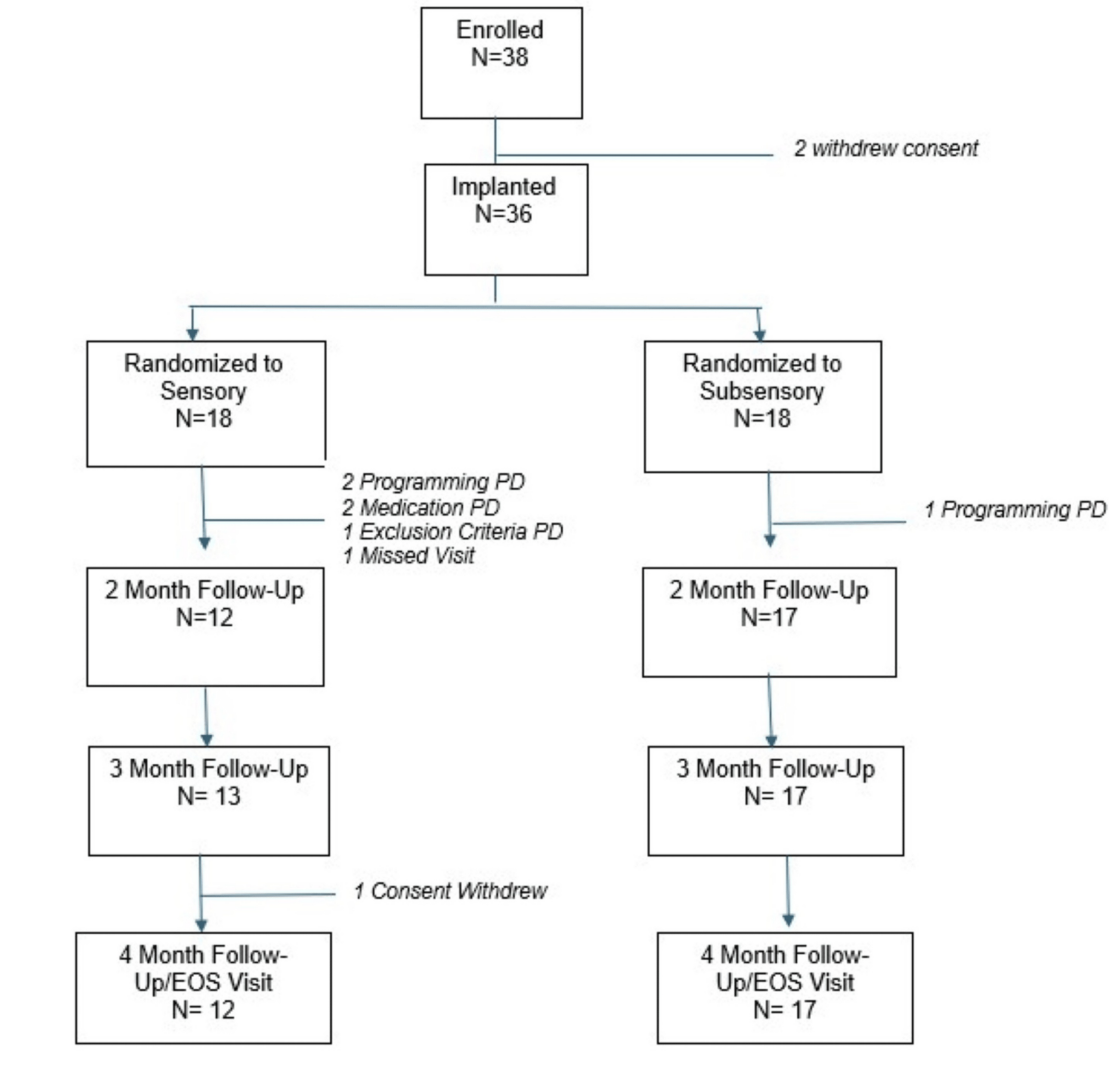
Subject Disposition PD: protocol deviation; EOS: end of study

Thirty-six subjects met eligibility criteria and were implanted with an eCoin ITNS at five centers in the United States. Thirty-five patients completed their three-month follow-up visit. During activation, three subjects experienced protocol deviations related to programming into the assigned treatment arm and were excluded from the per-protocol analysis population. In addition, two subjects experienced a protocol deviation related to OAB medication, and one subject experienced a protocol deviation for exclusion criteria and was also excluded from the per-protocol analysis population. Since the objective of the study is to explore the effect of sensory and subsensory amplitude settings, the per-protocol population is the primary analysis population in order to assess subjects who did not have deviations from the assigned amplitude settings. Figure 2 shows the subject disposition.

Table 2 shows the baseline demographics of the per-protocol population separated by treatment arms. Subjects, 27 (90%) women and 3 (10%) men, ranged in age from 33 to 88 (mean ± SD = 68 ± 11). Overall, 27 (90%) were White patients, 1 (3%) was a Black patient, and 1 (3%) was an Asian patient. Additionally, 1 (3%) was a Hispanic/Latino patient, while 27 (90%) were non-Hispanic/Latino patients. OAB treatment history is displayed in Table 3. Seven (23%) patients received PTNS therapy prior, and all the 30 patients received oral OAB medications, three (10%) received onabotulinumtoxinA, and two (7%) received transcutaneous electrical nerve stimulation (TENS) therapy.

**Table 2:** Demographic Characteristics of the Per-Protocol Population

Patient Demographics Per Protocol	Total (N=30)			Sensory (N=13)			Sub-sensory (N=17)		
Age, years at enrollment (N)	30			13			17		
Mean (SD)	68	(11)		69	(9)		66	(12)	
Range (min, max)	55	(33, 88)		32	(56, 88)		49	(33, 82)	
Quartiles (25th, median, 75th)	63	68	74	64	66	72	60	71	74
Sex, n (%)									
Female	27	(90)		12	(92)		15	(88)	
Male	3	(10)		1	(8)		2	(12)	
Smoking Status, n (%)									
Current smoker	0	0		0	0		0	0	
Race, n (%)									
White	27	(90)		13	(100)		14	(82)	
Black	1	(3)		0	0		1	(6)	
Asian	1	(3)		0	0		1	(6)	
Hawaiian	0	0		0	0		0	0	
Prefer Not to Answer	1	(3)		0	0		1	(6)	
Ethnicity, n (%)									
Hispanic/Latino	1	(3)		0	0		1	(6)	
Non-Hispanic/Latino	27	(90)		0	0		1	(6)	
Prefer Not to Answer	2	(7)		0	0		2	(12)	

**Table 3:** History of OAB Treatment in Subjects OAB: overactive bladder; PTNS: percutaneous tibial nerve stimulation; TENS: transcutaneous electrical nerve stimulation

Response to Prior Therapy	Total n (%) (N=30)
PTNS therapy	7 (23)
PTNS therapy response	3 (43)
TENS therapy	2 (7)
TENS therapy response	2 (100)
OAB therapy	30 (100)
OAB therapy response	0 (0)
Botox therapy	3 (10)
Botox therapy response	3 (100)

UUI outcomes

**Figure 3:**
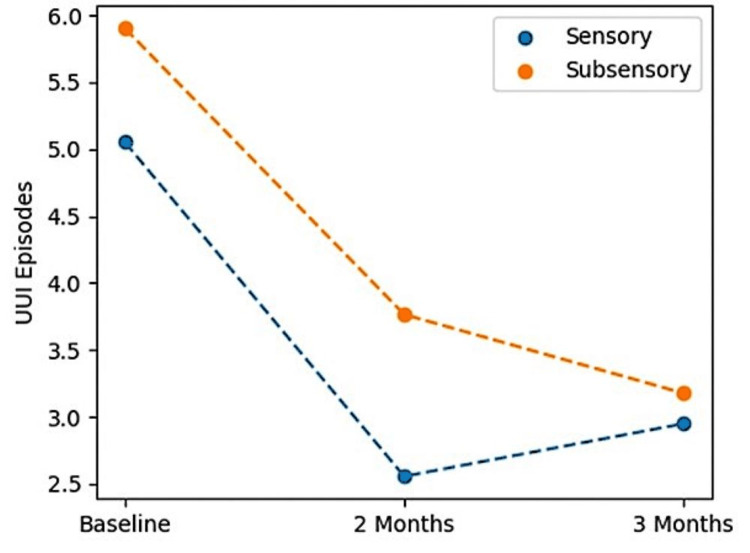
Average UUI Episodes Over Time by Treatment Arm UUI: urgency urinary incontinence

For the primary analysis at three months post-activation, the pooled mean reduction of UUI episodes per day was 2.46, with a mean reduction of 2.1 for the sensory arm and a mean reduction of 2.73 for the sub-sensory arm. At three months, three subjects were completely dry with 0 leaks reported on a three-day voiding diary, all three subjects were in the sub-sensory group. Table 4 summarizes data from the per-protocol analysis set. Average UUI episodes over time, with sensory and sub-sensory settings, are shown in Figure 3.

**Table 4:** Reduction in UUI Episodes for Pooled, Sensory, and Sub-sensory Groups in the Per-Protocol Population, Presented As Change From Baseline (CFB) at Two-, Three-, and Four-Month Follow-Up UUI: urgency urinary incontinence; BL: baseline

	Pooled			Sensory			Sub-sensory		
UUI episodes at BL									
N	30			13			17		
Mean (SD)	5.53	(2.62)		5.05	(2.01)		5.9	(3.01)	
Quartiles (25^th^, median, 75^th^)	3.42	4.83	7.25	3.33	5.0	6.33	3.67	4.67	7.67
Range (min, max)	11.67	(2.0)	(13.67)	6.0	(2.0)	(8.0)	11.0	(2.67)	(13.67)
UUI CFB at two months									
N	29			12			17		
Mean (SD)	-2.39	(2.3)		-2.75	(1.9)		-2.14	(2.58)	
Quartiles (25th, median, 75th)	-3.67	-2.0	-1.33	-3.67	-2.17	-1.92	-3.67	-2.0	-0.33
Range (min, max)	9.0	(-6.33)	(2.67)	7.0	(-6.33)	(0.67)	9.0	(-6.33)	(2.67)
UUI CFB at three months									
N	30			13			17		
Mean (SD)	-2.46	(2.07)		-2.1	(2.42)		-2.73	(1.8)	
Quartiles (25^th^, median, 75^th^)	-4.0	-2.5	-1.33	-2.67	-1.67	-1.33	-4.0	-2.67	-2.0
Range (min, max)	8.67	(-6.67)	(2.0)	8.67	(-6.67)	(2.0)	6.33	(-5.33)	(1.0)
UUI CFB at four months				Unblinded			Unblinded		
N	30			-			-		
Mean (SD)	-2.8	(2.31)		-			-		
Quartiles (25^th^, median, 75^th^)	-4.17	-2.83	-1.67	-			-		
Range (min, max)	10.33	(-7.33)	(3.0)	-			-		

Quality of life

Table 5 summarizes the secondary objective change from baseline in the patient-reported quality of life measures as reported by the HRQL subset of the OABq. At two months and three months post-activation, the analysis shows an increase in HRQL in pooled data and the sensory and sub-sensory treatment arms.

**Table 5:** Quality of Life for Per-Protocol Population HRQL: health-related quality of life; BL: baseline; CFB: change from baseline

	Pooled			Sensory			Sub-sensory		
HRQL score at BL									
N	30			13			17		
Mean (SD)	55.31	(20.56)		58.15	(18.8)		53.13	(22.13)	
Quartiles (25^th^, median, 75^th^)	45.4	58.0	70.0	51.2	60.8	71.2	43.2	55.2	66.4
Range (min, max)	81.6	(13.6)	(95.2)	70.4	(14.4)	(84.8)	81.6	(13.6)	(95.2)
HRQL scores: CFB at two months									
N	29			12			17		
Mean (SD)	24.06	(22.8)		25.87	(21.6)		22.78	(24.19)	
Quartiles (25^th^, median, 75^th^)	6.4	26.4	39.2	14.6	26.0	33.2	5.6	26.4	40.0
Range (min, max)	99.2	(-23.2)	(76.0)	84.8	(-8.8)	(76.0)	88.0	(-23.2)	(64.8)
HRQL scores: CFB at three months									
N	30			13			17		
Mean (SD)	20.67	(23.67)		18.15	(22.63)		22.59	(24.95)	
Quartiles (25^th^, median, 75^th^)	3.4	18.4	40.2	3.2	18.4	28.8	4.0	18.4	41.6
Range (min, max)	91.2	(-21.6)	(69.6)	81.6	(-14.4)	(67.2)	91.2	(-21.6)	(69.6)
HRQL scores: CFB at four months				Unblinded			Unblinded		
N	30			-			-		
Mean (SD)	27.73	(25.97)		-			-		
Quartiles (25^th^, median, 75^th^)	7.0, 26.0, 41.6	26.0	41.6	-			-		
Range (min, max)	97.6	(-14.4)	(83.2)	-			-		

Patient satisfaction survey

**Figure 4:**
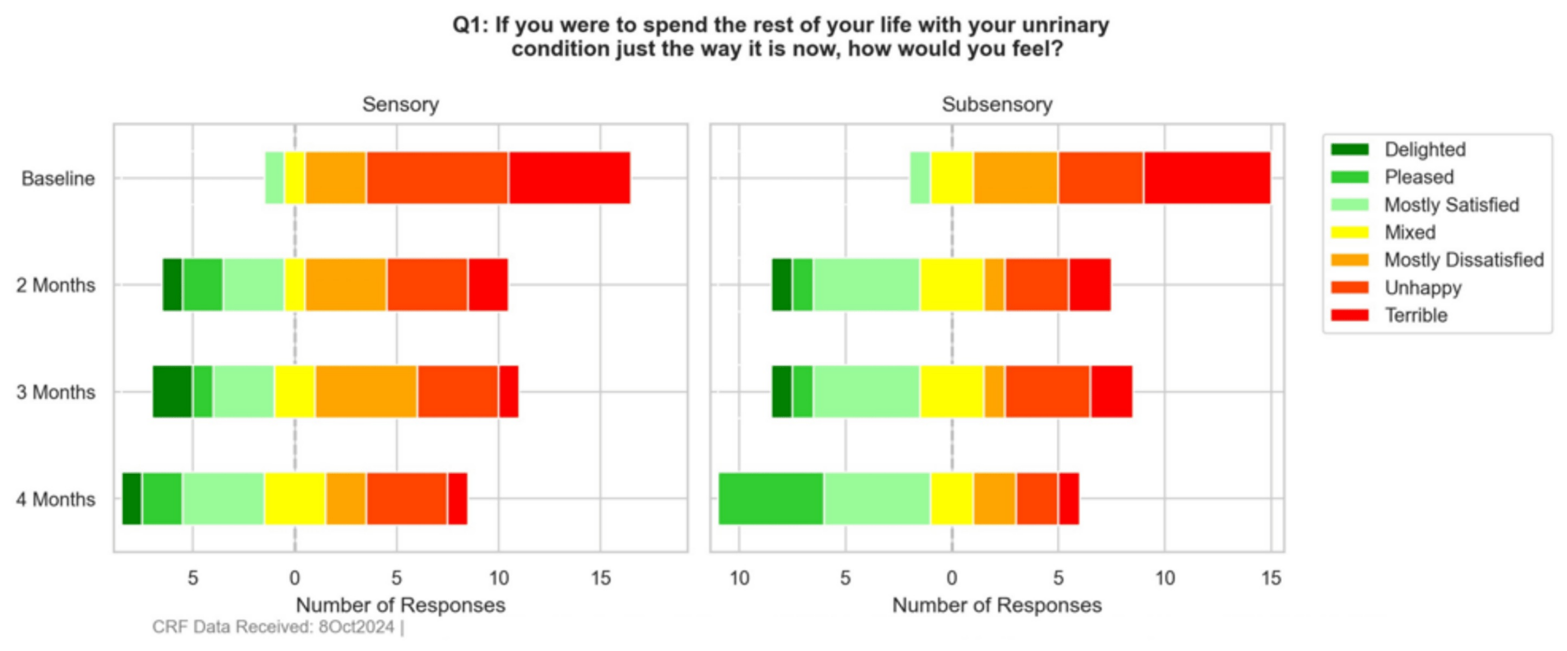
Patient Satisfaction Survey - Question 1 (Per-Protocol, All Available Data)

**Figure 5:**
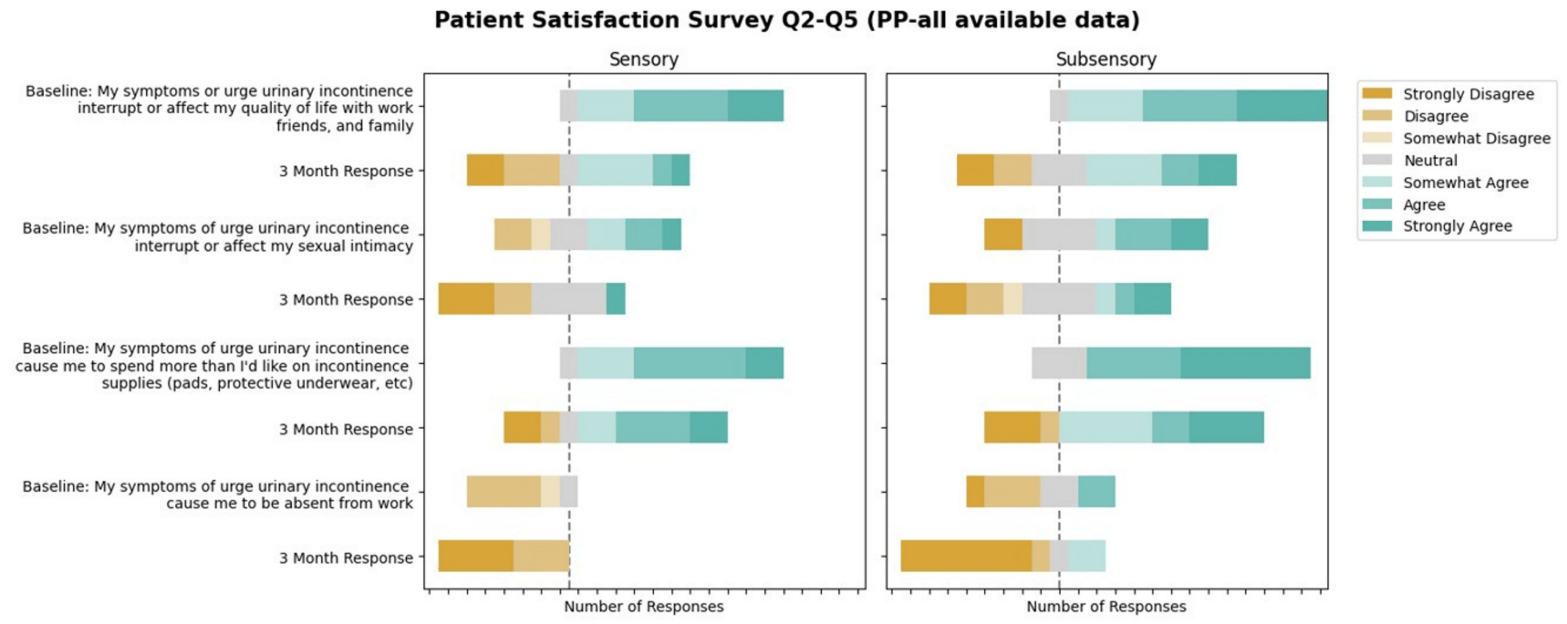
Patient Satisfaction Survey - Questions 2 to 5 (Per-Protocol, All Available Data)

The patient satisfaction survey results showed improvement in satisfaction among the sensory and sub-sensory treatment arms, as displayed in Figures 4-5.

## Discussion

OAB and UUI represent significant burdens on individuals' quality of life, affecting both men and women worldwide. Despite the availability of various treatment modalities, a substantial proportion of patients continue to experience bothersome symptoms, highlighting the need for further research into effective therapeutic options. ITNS has emerged as a promising intervention for refractory cases, offering FDA-approved relief for patients with UUI [9-12].

Prior investigations have suggested that optimizing stimulation parameters, particularly at sensory and sub-sensory levels, may modulate brain activation patterns, potentially enhancing treatment outcomes [13-16]. Building upon this premise, our research endeavors explore the impact of sensory and sub-sensory programming using the FDA-approved eCoin ITNS in reducing UUI episodes. By assessing the effectiveness of different programming methodologies, we aim to optimize therapy efficacy and extend the longevity of neurostimulator devices, thereby improving patient outcomes and satisfaction.

The eCoin ITNS represents a significant advancement in the field of neuromodulation for urinary dysfunction. Its fully implantable design offers a convenient and minimally invasive treatment option for patients suffering from UUI. The device can effectively modulate the neural pathways involved in bladder control by emitting a dome-shaped electrical field, thereby mitigating urgency, frequency, and incontinence symptoms. It delivers automatic 30-minute treatment sessions that require no patient intervention, further enhancing its ease of use, patient compliance, and efficiency in managing these symptoms.

The ESSENCE study showed, through a double-blind, RCT conducted across multiple centers in the United States, that patients with sub-sensory amplitude settings achieved a 2.73 reduction in UUI episodes/day, and patients with sensory amplitude settings achieved 2.1 per day at three months. It should be noted that comparative testing between sensory and sub-sensory groups was not conducted in accordance with the study design. This study, as an RCT, focused primarily on the efficacy of both sensory and sub-sensory settings but was not initially powered for a head-to-head comparison between these two groups.

Comparative statistical analysis between groups, such as a non-inferiority analysis, would require a significantly larger sample size to achieve robust, statistically significant results.

At two months, the results showed a numerically greater reduction in the sensory group with a reduction of 2.75 UUI episodes per day in the sensory group compared to 2.14 in the subsensory group, while the sub-sensory group showed a numerically greater reduction at three months. It is hypothesized that the treatment effect is slower in the subsensory group.

The observed delayed effect in the sub-sensory group compared to the sensory group may be attributed to differences in neural activation thresholds and mechanisms associated with sensory and sub-sensory stimulation. In neuromodulation therapy, the activation of neural pathways depends heavily on reaching the sensory threshold, which is typically characterized by recognizable stimulation that directly impresses a broader range of afferent nerve fibers involved in bladder control and sensation. By contrast, sub-sensory settings operate below this threshold, which may result in slower, more gradual neural adaptation and potentially distinct patterns of central nervous system engagement. Without the stronger, immediate stimulation associated with sensory-level activation, the sub-sensory group may experience a more gradual buildup in therapeutic effects as the neural circuits responsible for bladder control adapt to continuous, low-level input.

This effect may be explored further in future studies. The treatment effect was maintained at four months which showed a pooled mean reduction of 2.8 UUI episodes per day. These findings are similar to the findings published by Elterman et al., which assessed sensory and sub-sensory programming in SNM where patients with sub-sensory amplitude settings at 50% and 80% of sensory threshold demonstrated a reduction in UI episodes of -3.0 (95% CI: -4.4 to -1.7) and -2.9 (95% CI: -4.7 to -1.2) UI episodes/day, respectively and patients with amplitude settings at the sensory threshold had a reduction of -3.6 (95% CI: -5.2 to -1.9) UI episodes/day [6].

These findings show improvements in UUI episodes, quality of life, and patient satisfaction, offering important insights with potential impacts on patients. Using sub-sensory program settings may improve patient comfort while achieving the desired reduction in UUI. Notably, using sub-sensory amplitude settings extends the device's longevity, as the lifespan of the device is directly related to the amplitude setting. In fact, sub-sensory stimulation may increase device usage by up to eight years, adding years of life to the device compared to sensory levels.

By prolonging the device’s longevity, patients may experience fewer replacement procedures, a clear benefit for both their comfort and quality of life. Additionally, sub-sensory settings can minimize or even eliminate the need for device setting adjustments over the life of the device, which is a notable advantage for both the patient and the physician. Patients may avoid regular office visits to reprogram settings for comfort adjustments or to change the timing of stimulation, tailoring it to their daily routines. This reduction in required adjustments not only further elevates the quality of life for patients but also lessens the engagement and follow-up demands on physicians and technicians. In summary, sub-sensory stimulation offers a holistic benefit: enhancing battery longevity while optimizing both patient comfort and provider efficiency.

The ESSENCE study included an End of Study Survey at the four-month follow-up, prompting participants to reflect on their experience with eCoin. Among the 35 subjects who completed the four-month visit, seven had previously achieved successful outcomes with PTNS, TENS, OAB medications, or Botox. Of these, five (71%) expressed a preference for eCoin over their prior treatments, while two (29%) remained neutral. Additionally, six out of seven (86%) reported that they would recommend eCoin to family and friends and found the device easy to understand and use. Prior to starting eCoin therapy, patients were asked about factors influencing treatment adherence for UUI at their screening visit. Participants cited effectiveness, treatment burden, risk, and concerns regarding treatment appearance as their primary considerations.

Similar to our study, a study conducted by Perez-Martinez et al. evaluated the efficacy of six sessions of PTNS for treating OAB symptoms in men and women, compared to the standard 12 sessions. The results demonstrated significant improvements in OAB symptoms and quality of life at the six-month follow-up. Both studies share a common goal of optimizing neurostimulation parameters to enhance the patient experience. While the PTNS study focused on reducing the number of treatment sessions to minimize the patient burden, our study aimed to explore sub-sensory programming parameters to improve battery longevity and reduce patient discomfort. In both cases, the focus remains on making neuromodulation therapy more efficient and patient-centered [17].

A limitation of this study is the lack of a statistically significant comparison between groups. Though both groups show a reduction in UUI, improvement in quality of life, and patient satisfaction, it is unknown if the effects observed in the sub-sensory group are non-inferior to the sensory group.

## Conclusions

This study demonstrates that both sensory and sub-sensory amplitude settings of the eCoin ITNS are effective in reducing UUI episodes and improving the quality of life for patients. The data support that sub-sensory programming offers additional advantages, including enhanced patient comfort and extended device longevity, potentially delaying the need for replacement procedures. This has significant implications for patient satisfaction and long-term treatment adherence, as it may reduce the frequency of device-related medical visits and adjustments, ultimately improving overall patient experience and clinical efficiency.

While the study design lacked sufficient power to demonstrate statistical significance and establish the non-inferiority of the sub-sensory group compared to the sensory group, the observed data trends support the potential benefits of sub-sensory programming. However, further research is necessary to solidify these findings and confirm the non-inferiority of sub-sensory settings compared to sensory programming. Larger-scale studies with sufficient power for comparative analysis could better outline the differences between these two approaches. Nevertheless, the results highlight the promising potential of sub-sensory stimulation as a refined strategy in ITNS therapy, balancing effective UUI management with long-term patient-centered care.
